# Effect of targeted coagulopathy management and 5% albumin as volume replacement therapy during lung transplantation on allograft function: a secondary analysis of a randomized clinical trial

**DOI:** 10.1186/s12890-023-02372-0

**Published:** 2023-03-09

**Authors:** Jaromir Vajter, Jiri Vachtenheim, Zuzana Prikrylova, Jan Berousek, Tomas Vymazal, Robert Lischke, Archer Kilbourne Martin, Miroslav Durila

**Affiliations:** 1 Department of Anesthesiology and Intensive Care Medicine, Second Faculty of Medicine, Charles University and University Hospital Motol, Prague, Czech Republic; 2 Prague Lung Transplant Program, 3rd Department of Surgery, First Faculty of Medicine, Charles University and University Hospital Motol, Prague, Czech Republic; 3 Division of Cardiovascular and Thoracic Anesthesiology, Mayo Clinic College of Medicine and Science, Jacksonville, FL USA

**Keywords:** Lung transplantation, Anesthetic management, Rotational thromboelastometry, Volume replacement therapy, 5% albumin

## Abstract

**Background:**

Primary graft dysfunction (PGD) after lung transplantation (LuTx) contributes substantially to early postoperative morbidity. Both intraoperative transfusion of a large amount of blood products during the surgery and ischemia–reperfusion injury after allograft implantation play an important role in subsequent PGD development.

**Methods:**

We have previously reported a randomized clinical trial of 67 patients where point of care (POC) targeted coagulopathy management and intraoperative administration of 5% albumin led to significant reduction of blood loss and blood product consumption during the lung transplantation surgery. A secondary analysis of the randomized clinical trial evaluating the effect of targeted coagulopathy management and intraoperative administration of 5% albumin on early lung allograft function after LuTx and 1-year survival was performed.

**Results:**

Compared to the patients in the control (non-POC) group, those in study (POC) group showed significantly superior graft function, represented by the Horowitz index (at 72 h after transplantation 402.87 vs 308.03 with *p* < 0.001, difference between means: 94.84, 95% CI: 60.18–129.51). Furthermore, the maximum doses of norepinephrine administered during first 24 h were significantly lower in the POC group (0.193 vs 0.379 with *p* < 0.001, difference between the means: 0.186, 95% CI: 0.105–0.267). After dichotomization of PGD (0–1 vs 2–3), significant difference between the non-POC and POC group occurred only at time point 72, when PGD grade 2–3 developed in 25% (*n* = 9) and 3.2% (*n* = 1), respectively (*p* = 0.003). The difference in 1-year survival was not statistically significant (10 patients died in non-POC group vs. 4 patients died in POC group; *p* = 0.17).

**Conclusions:**

Utilization of a POC targeted coagulopathy management combined with Albumin 5% as primary resuscitative fluid may improve early lung allograft function, provide better circulatory stability during the early post-operative period, and have potential to decrease the incidence of PGD without negative effect on 1-year survival.

**Trial registration:**

This clinical trial was registered at ClinicalTrials.gov (NCT03598907).

## Background

Lung transplantation (LuTx) remains the ultimate treatment for end-stage lung disease refractory to optimized medical therapy. Post-implantation, long-term outcomes are impaired by ongoing medical factors including chronic lung allograft dysfunction (CLAD). CLAD (and its phenotypes) represents a major complication that limits the 5-year survival to approximately 55% [1–3]. CLAD develops as a result of various alloimmune-dependent and alloimmune-independent graft injuries and dysregulated repair processes. Primary graft dysfunction (PGD) has been identified as an important risk factor for CLAD development [4, 5].

PGD is defined by the presence of diffuse pulmonary opacities on thoracic imaging and various levels of hypoxemia without other identifiable causes developing in the first 72 h after lung allograft reperfusion [6]. Its clinical course in the most severe form resembles acute respiratory distress syndrome (ARDS) and is considered to be one of the most important causes of early death after transplantation, with an incidence of PGD reported between 10 and 25% [7]. Furthermore, patients who develop PGD also show significantly worse long-term outcomes [8, 9]. Although the pathophysiology of PGD is not completely understood and multifactorial, several intraoperative anesthetic risk factors have been described within the literature [10]. For instance, intraoperative administration of blood products is associated with a strong negative influence on PGD development and outcome in lung transplant recipients [11]. Moreover, a large volume of intraoperative fluids and red blood cells (RBCs) significantly correlates with the development of PGD grade 3. Therefore, the limitation of intraoperative fluid and blood product administration may reduce the risk for the development of PGD grade 3 and thus improve early postoperative morbidity and mortality after LuTx [12].

A reduction in blood loss during surgery and corresponding decrease in intraoperative transfusion of blood products can be achieved by utilizing intraoperative point of care (POC) targeted bleeding/coagulopathy management strategies such as rotational thromboelastometry (ROTEM), platelet function analyzer (PFA) or multiple electrode platelet aggregometry [13, 14]. The use of these approaches has been reported in studies on cardiac surgery and liver transplantation [15–17]. Previously, we reported that POC-targeted coagulopathy management decreases perioperative blood loss and consumption of RBCs and fresh frozen plasma (FFP) during LuTx [18].

Despite the abovementioned reduction in blood product transfusion, ongoing fluid resuscitation is necessary to maintain normovolemia during LuTx surgery. A few studies have described the potential benefits of 5% albumin solution administration for the treatment of patients with ARDS and during cardiac surgery [19, 20]. However, data on the role of perioperative 5% albumin administration and its effect on lung allograft function are lacking, and further investigation is highly needed.

We present a secondary analysis of our randomized clinical trial evaluating the effect of POC coagulopathy management and intraoperative administration of 5% albumin as primary resuscitative fluid during LuTx surgery on early lung allograft function, incidence of PGD, and 1-year survival.

## Methods

### Study design overview, surgical strategy and outcomes

A secondary analysis of the Point of Care Management of Coagulopathy in Lung Transplantation trial (NCT03598907) was performed. This study was a single-site, prospective randomized controlled trial that examined the utilization of perioperative POC-targeted coagulopathy management in conjunction with 5% albumin solution and their effect on perioperative blood loss and consumption of blood products during LuTx. This study was approved by the institutional ethics committee (reference number EK-1402/17) and was registered in the clinical trial database at ClinicalTrials.gov (identifier number NCT03598907) prior to patient enrollment. All patients provided written informed consent for participation in the study before the LuTx procedure.

As this was a pilot study, the projected number of patients to be recruited was estimated at 120 (planned for 4 years), and an a priori power analysis was not performed in this case. An interim analysis was planned after evaluation of approximately 60 patients (after 2 years). Patients were primarily randomized to two study groups – POC group and non-POC group. The perioperative anesthesia management strategy used for both the non-POC group and POC group has been described previously [18]. Importantly, in the non-POC group, perioperative bleeding, coagulopathy management and volume replacement therapy was managed according to the clinical experience of the anesthesiologist consisting of blood loss monitoring and subjective optical inspection of the surgical field hemostasis without using POC targeted coagulopathy management or laboratory analysis.

Intravascular volume in this group was replaced with balanced crystalloid, non-albumin colloidal solutions such as 6% hydroxyethyl starch or 4% succinylated gelatin, and FFP. Median volume of balanced crystalloid solution and non-albumin colloidal solutions was 1000 ml (IQR 512.5; 987.5 – 1500) and 775 ml (IQR 500; 500 – 1000), respectively. Triggers to volume replacement in both POC and non-POC groups included circulatory stability expressed by dose of norepinephrine (µg/kg/min) and transesophageal echocardiography (TEE) assessment of decreased cardiac preload by left ventricular fractional area change in transgastric mid-papillary short axis view at 50% calculated veno-arterial extracorporeal membrane oxygenation (VA ECMO) flow. However, due to the limitations described of TEE as a sole intraoperative monitor of systemic volume during VA ECMO, monitors aiding in the assessment of ongoing resuscitation also included urine output and maintenance of pulsatility within systemic and pulmonary arterial waveforms [21].

**Fig. 1:**
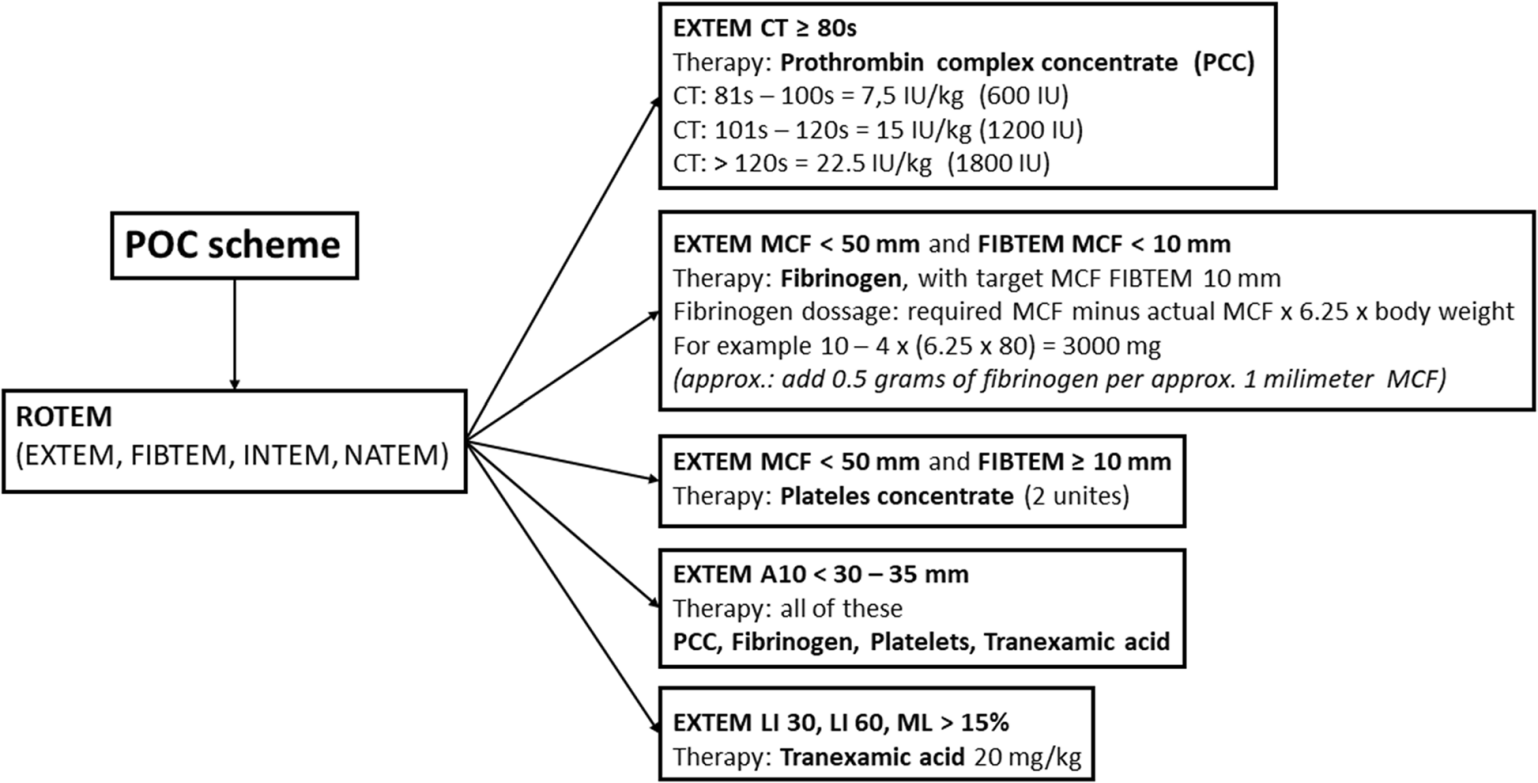
ROTEM protocol for the diagnosis of coagulopathy and goal-directed therapy using EXTEM, FIBTEM, and APTEM. Abbreviations: A10: Amplitude at 10 min; CT: clotting time; IU: international unit; LI30, LI60: lysis index at 30 and 60 min, MCF: maximum clot firmness; ML: maximum lysis. Previously published in Durila M, Vajter J, Garaj M, Pollert L, Berousek J, Vachtenheim J, Jr., et al. Rotational thromboelastometry reduces blood loss and blood product usage after lung transplantation. J Heart Lung Transplant. 2021;40(7):631–41.^18^

In the POC group, perioperative bleeding and coagulopathy were managed according to the POC methods performed at the beginning of the surgery, after reperfusion of the first implanted lung and at the end of the surgical procedure (Fig. 1). In this group, a 5% albumin solution was exclusively used for intravascular volume replacement therapy to maintain normovolemia. Median volume of 5% albumin administered was 1750 ml (IQR 500; 1500—2000). The laboratory trigger for RBCs administration in both patient groups was haemoglobin level of 100 g/l. The surgical strategy, lung procurement and ECMO support handling adhered to the methods previously described by the Vienna Lung Transplant Group [22]. At our institution, intraoperative ECMO support is routinely used pre-emptively in the majority of cases during LuTx and only a smaller number of cases LuTx are performed without any extracorporeal life support, as this is purely at the discretion of the transplanting surgeon. In the POC group, the intraoperative ECMO circuit was primed with albumin. The surgical procedural aspects remained consistent throughout the study period and did not differ in either group.

Primary outcome for this secondary analysis was PGD development and grading during the first 72 h after lung transplantation. Measures of Horowitz index (P/F ratio; defined as arterial oxygen pressure (P_a_O_2_) in mmHg divided by fraction of inspired oxygen (F_i_O_2_) in %) and serum albumin levels in both groups before and after lung transplantation were analyzed. Circulatory stability status characterized by the maximum level of norepinephrine administered during the first 24 h after LuTx was analyzed, with norepinephrine administration based on mean arterial pressure. Postoperative duration of mechanical ventilation and length of intensive care unit (ICU) stay were recorded. Secondary outcome for this analysis was 1-year survival in both groups.

### Patient selection and enrollment

Patient selection and eligibility criteria for the current secondary analysis mirrored that of the Point of Care Management of Coagulopathy in Lung Transplantation trial and included patients who underwent LuTx at the University Hospital Motol between January 2018 and June 2020 [18]. The exclusion criteria were electively prolonged postoperative ECMO (patients with idiopathic pulmonary hypertension or preoperatively known severe secondary pulmonary hypertension on basis of underlying disease that were preoperatively identified to require intended ECMO prolongation leading automatically to classification as PGD grade 3, as this would result in negative impact on interpretation of the study), pediatric recipients, single-lung transplantations, retransplantations, heart–lung transplantations, and transplantations requiring cardiopulmonary bypass for technical reasons (concomitant cardiac surgery).

**Fig. 2:**
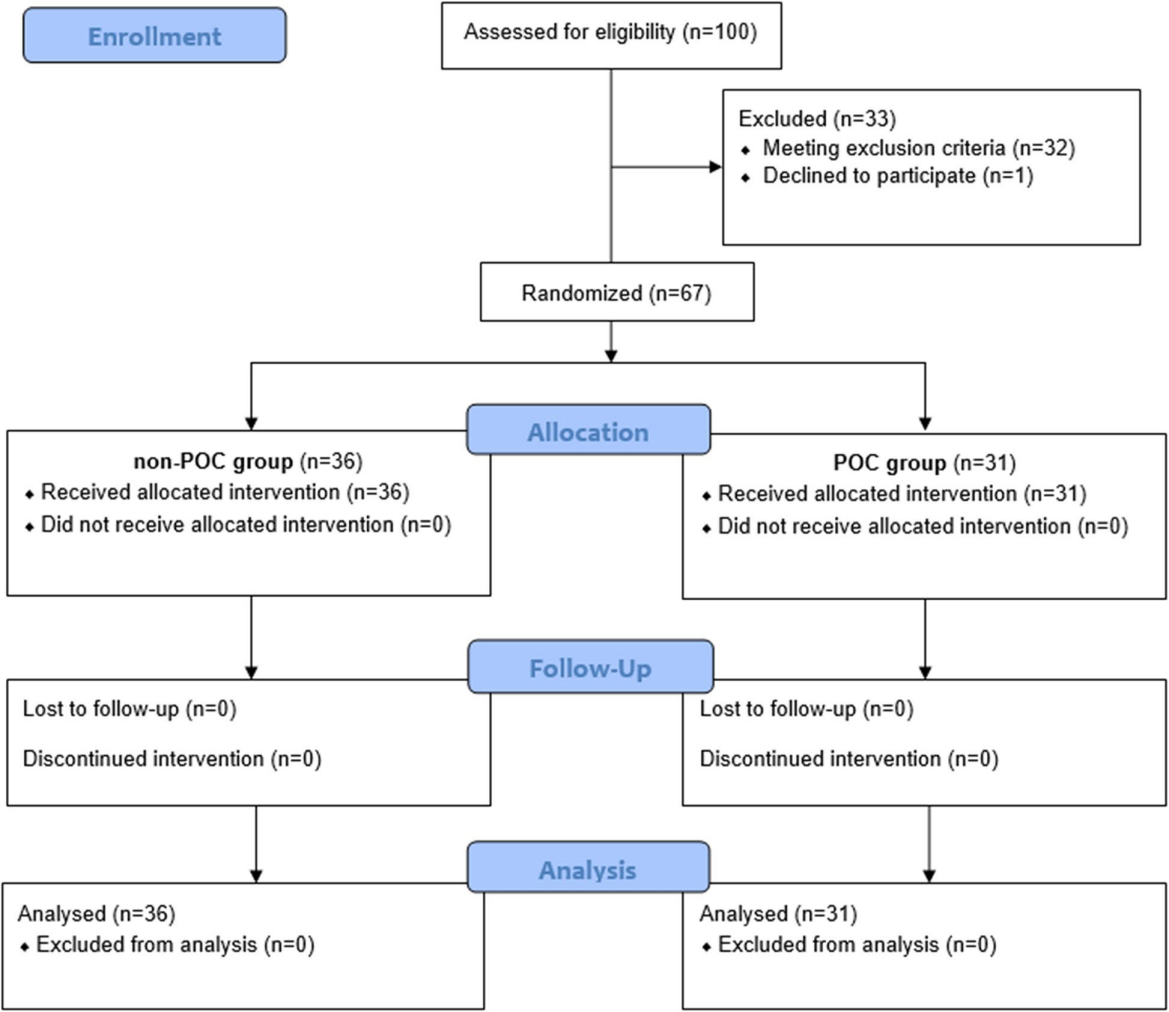
Flow chart of the study population

Randomization and detailed description of two randomized groups were described previously [18]. In the first group (POC group), 31 patients were analyzed, and in the second group (non-POC group), 36 patients were analyzed. A flow diagram based on the Consolidated Standards of Reporting Trials (CONSORT) is displayed in Fig. 2.

### PGD definition

The definition of PGD was based on the latest International Society for Heart and Lung Transplantation (ISHLT) recommendation and was recorded 2 h after ICU admission (time 0) and then 24 h (time 24), 48 h (time 48) and 72 h (time 72) after LuTx [6]. Chest radiographs assessment was consistent with the methods previously described by the Vienna Lung Transplant Group [22].

### Statistical analysis

Statistical analyses were performed with R statistical software, version 3.4.4 (available online at http://www.r-project.org/). A *p*-value of 0.05 was considered statistically significant. PGD grade 1 is questionably relevant clinically, therefore PGD grades were dichotomized into two groups and analyzed as follows: PGD 0—1 vs PGD 2—3. The Fischer exact test was performed with the data from each time point (0, 24, 48, 72 h) to analyze the association of dichotomized PGD in both groups. Serum albumin levels in both groups were measured before and after LuTx. Due to technical reasons, the preoperative serum albumin levels in 15 patients were not measured (9 patients in the non-POC group and 6 patients in the POC group). Postoperative serum albumin levels were completed for all patients in the study cohort.

The Horowitz index was calculated at each tracked time point (0, 24, 48, 72 h), and the measured values were evaluated with Welch’s two-sample t-test. The maximum level of norepinephrine (µg/kg/min) during the first 24 h after LuTx was compared in both groups using Welch’s two-sample t-test. Postoperative ICU stay and mechanical ventilation duration was recorded and analyzed by Wilcoxon tests. Moreover, 1-year survival in both study groups was followed and survival rates were compared using log-rank tests.

## Results

### Study patients and study flow

Patients were recruited during the period from January 2018 to June 2020, and based on the exclusion criteria, a total of 33/100 patients were excluded from the study. The non-POC group and POC group ultimately consisted of 36 and 31 patients, respectively. At this point, interim statistical analysis was performed, and the study was preliminarily terminated by the institutional review board because the results were significantly in favor of the POC approach, as significant decrease in perioperative blood loss and related decrease in blood products consumption was observed among the POC study group [18]. In the POC group and non-POC group, the mean blood loss in the operating room was 682 ml ± 399 and 1043 ml ± 547, respectively (*p* = 0.003). Mean value of RBCs units administered in the operating room was 0.83 ± 1.15 in the POC group and 1.05 ± 1.45 in the non-POC group (*p* = 0.506). Mean value of FFP units administered in the operating room was 0.00 in the POC group and 4.08 ± 2.89 in the non-POC group (*p* < 0.001) [18].

Patients in the non-POC group were significantly older than patients in POC group (56.22 ± 9.05 vs 45.69 ± 16.54 years, *p* = 0.002), as the proportion of younger patients with cystic fibrosis was significantly higher in the POC group (32.3%, *n* = 10 vs 5,6%, *n* = 2; *p* = 0.005). The use of intraoperative ECMO support compared to off pump approach was higher in non-POC group, although the difference was not statistically significant (86%, *n* = 31 vs 67.7%, *n* = 21; *p* = 0.07). However, mean pulmonary arterial pressure, that would signalize higher degree of disease severity and complexity did not differ significantly between recipients in non-POC and POC group. Detailed preoperative and intraoperative characteristics of the recipients have been reported previously and are presented with permission in Table 1 together with donor characteristics [18]. There were no statistically significant differences in the donor variables between the non-POC and POC group. In POC group, only 1 out of 31 patients received organ from donation after circulatory death (DCD) donor and no organ from DCD donor was utilized in non-POC group. No organ from expanded criteria donor was utilized (not shown in Table 1). Importantly, no case of graft dysfunction at the end of the surgery that would require ECMO prolongation occurred in either study group.

**Table 1:** Recipient and donor characteristics

**Recipient characteristics variable**	**non-POC group (*n*** **= 36)**	**POC group (*n*** **= 31)**	***p*-value**
Male sex	25 (69%)	20 (64.5%)	0.67
Age (years; mean ± SD)	56.22 ± 9.05	45.69 ± 16.54	0.002
Weight (kg; mean ± SD)	76.64 ± 18.21	67.48 ± 16.51	0.036
Height (cm; mean ± SD)	173.81 ± 9.72	169.55 ± 9.57	0.08
Body mass index (mean ± SD)	25.03 ± 4.04	23.25 ± 4.33	0.09
MPAP (mmHg; mean ± SD)	23.97 ± 6.05	25.58 ± 9.63	0.43
Transplant indication			
COPD	15 (41.6%)	12 (38.7%)	0.81
Pulmonary fibrosis	18 (50%)	9 (29%)	0.08
Cystic fibrosis	2 (5.6%)	10 (32.3%)	0.005
Sarcoidosis	1 (2.8%)	0	0.35
Intra-Operative recipient characteristics			
Thoracotomy			
Sternum sparing	5 (14%)	8 (25.8%)	0.22
Clamshell	31 (86%)	23 (74.2%)	0.22
Intraoperative ECMO	31 (86%)	21 (67.7%)	0.07
Ischemic time (min; mean ± SD)			
First lung	242.83 ± 40.23	243.52 ± 40.96	0.91
Second lung	353.69 ± 47.21	354.06 ± 58.02	0.24
**Donor characteristics variable**	**non-POC group (*n*** **= 36)**	**POC group (*n*** **= 31) **	***p*-value**
Male sex	23 (63.9%)	17 (54.8%)	0.45
Age (years; mean ± SD)	43.56 ± 18.84	43.81 ± 15.14	0.95
Weight (kg; mean ± SD)	71.67 ± 16.32	73.77 ± 18.12	0.62
Height (cm; mean ± SD)	170.92 ± 12.98	173.81 ± 9.95	0.32
Body mass index (mean ± SD)	25.13 ± 6.71	24.28 ± 5.26	0.57
Horowitz index (mmHg; mean ± SD)	468.56 ± 64.58	475.32 ± 63.83	0.67
Smoking history, *n* (%)	8 (22.2%)	5 (16.1%)	0.53
Cause of death, *n* (%)			
subarachnoid hemorrhage	5 (13.9%)	7 (22.6%)	0.35
intracerebral bleeding	9 (25%)	9 (29%)	0.71
trauma capitis	13 (36.1%)	11 (35.5%)	0.96
anoxic brain damage	7 (19.4%)	1(3.2%)	0.06
other	2 (5.6%)	3 (9.7%)	0.66

*Abbreviations*: *COPD* chronic obstructive pulmonary disease, *ECMO* extracorporeal membrane oxygenation, *MPAP* mean pulmonary arterial pressure, *POC* point of care, *SD* standard deviation. Previously published in Durila M, Vajter J, Garaj M, Pollert L, Berousek J, Vachtenheim J, Jr., et al. Rotational thromboelastometry reduces blood loss and blood product usage after lung transplantation. J Heart Lung Transplant. 2021;40(7):631–41.^18^

### Primary graft dysfunction evaluation

**Fig. 3:**
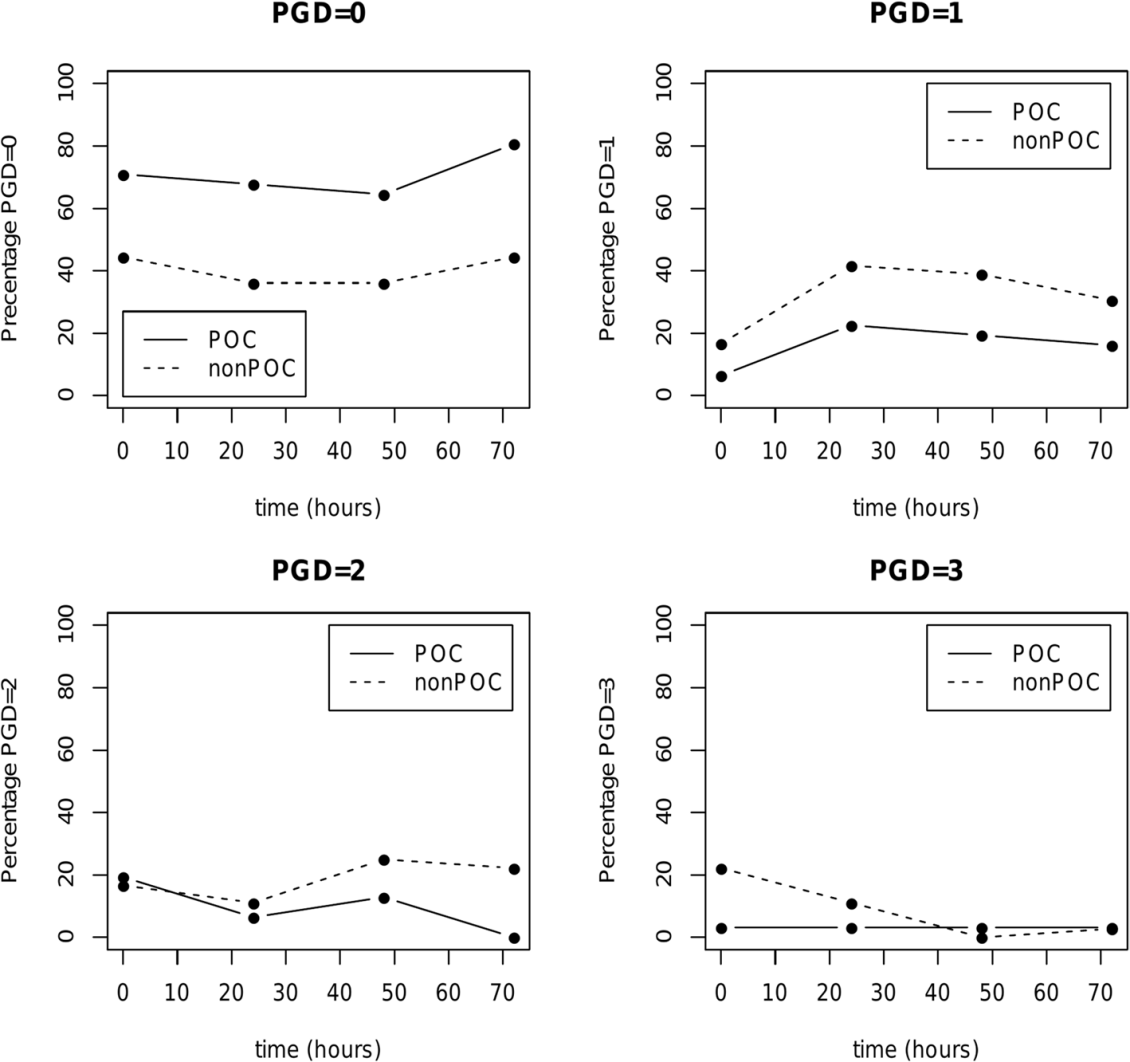
Incidence of primary graft dysfunction after lung transplantation at 0, 24, 48 and 72 h after surgery. Abbreviations: PGD: primary graft dysfunction; POC: point of care

The incidence of PGD development based on ISHLT criteria at each time point in the non-POC and POC groups is displayed in Table 2 and Fig. 3 [6]. No PGD (grade 0) was found significantly more frequently in the POC group at every tracked time point, although the overall difference in PGD (regardless of grade) was statistically significant only at time point 72. However, PGD grade 0 and even 1 are questionably relevant clinically, therefore PGD grades were further dichotomized and analyzed into two categories according to clinical relevance (PGD grade 0 – 1 vs PGD grade 2 – 3) and results are shown in Table 3. Significant difference between the non-POC and POC group occurred only at time point 72, when PGD grade 0 – 1 was observed in 75% (*n* = 27) and 96.8% (*n* = 30), respectively. At the same time point 72, PGD grade 2 – 3 developed in 25% (*n* = 9) and 3.2% (*n* = 1), respectively (*p* = 0.016). Of those 9 patients with PGD grade 2 – 3 in the non-POC group at time point 72, 8 patients had PGD grade 2 and 1 patient had PGD grade 3. There was no statistically significant difference in occurrence of PGD grade 3 between the non-POC and POC group at all tracked time points.

**Table 2:** Primary graft dysfunction development in both groups in four tracked time periods. Data are presented as n (%)

Time 0					
PGD grade	0	1	2	3	*p*-value
non-POC	16 (44.4)	6 (16.7)	6 (16.7)	8 (22.2)	0.048
POC	22 (70.9)	2 (6.5)	6 (19.4)	1 (3.2)	
Time 24					
PGD grade	0	1	2	3	*p*-value
non-POC	13 (36.1)	15 (41.7)	4 (11.1)	4 (11.1)	0.08
POC	21 (67.7)	7 (22.6)	2 (6.5)	1 (3.2)	
Time 48					
PGD grade	0	1	2	3	*p*-value
non-POC	13 (36.1)	14 (38.9)	9 (25)	0	0.052
POC	20 (64.5)	6 (19.4)	4 (12.9)	1 (3.2)	
Time 72					
PGD grade	0	1	2	3	*p*-value
non-POC	16 (44.4)	11 (30.6)	8 (22.2)	1 (2.8)	0.003
POC	25 (80.7)	5 (16.1)	0	1 (3.2)	

*Abbreviations*: *PGD* primary graft dysfunction, *POC* point of care

**Table 3:** In both patient groups, PGD grades were dichotomized and analyzed into two categories according to their clinical relevance (PGD grade 0 – 1 vs PGD grade 2 – 3). Data are presented as n (%)

Time 0			
PGD grade	non-POC	POC	*p*-value
0—1	22 (61.1)	24 (77.4)	0.19
2—3	14 (38.9)	7 (22.6)	
Time 24			
PGD grade	non-POC	POC	*p*-value
0—1	28 (77.8)	28 (90.3)	0.2
2—3	8 (22.2)	3 (9.7)	
Time 48			
PGD grade	non-POC	POC	*p*-value
0—1	27 (75)	26 (83.9)	0.55
2—3	9 (25)	5 (16.1)	
Time 72			
PGD grade	non-POC	POC	*p*-value
0—1	27 (75)	30 (96.8)	0.016
2—3	9 (25)	1 (3.2)	

*Abbreviations*: *PGD* primary graft dysfunction, *POC* point of care

### Horowitz index evaluation

**Fig. 4:**
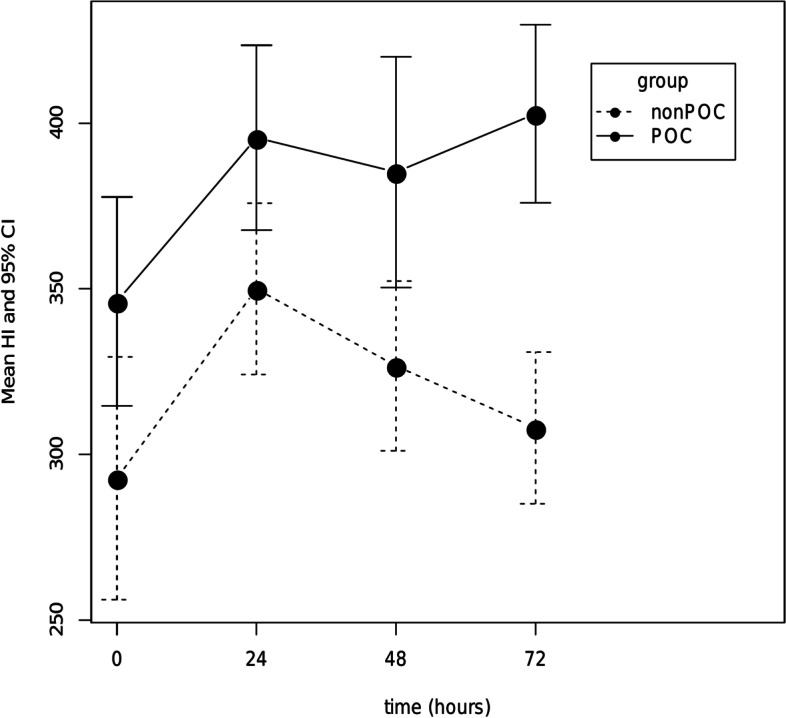
Horowitz index at each time point (0, 24, 48, 72 h) after lung transplant surgery. Values are presented as the mean and 95% CI. Abbreviations: HI: Horowitz index; CI: confidence interval; POC: point of care

Table 4 and Fig. 4 show the mean values of the Horowitz index at each time point (0, 24, 48, 72) in the non-POC group and the POC group. At all tracked time points, pulmonary graft function was significantly higher in the POC group, as indicated by the Horowitz index. It is of particular interest, that most significant difference between the groups occurred at time point 72, when the Horowitz index was 308.03 in the non-POC group vs 402.87 in the POC group (*p* < 0.001, difference between means: 94.84, 95% CI: 60.18–129.51).

**Table 4:** Horowitz index and its differences between the non-POC and POC group at each tracked time. The Horowitz index is defined as arterial oxygen pressure (P_a_O_2_) in mmHg divided by the fraction of inspired oxygen (F_i_O_2_) in %. Values are displayed together with the difference estimate and confidence intervals (CI) to illustrate the difference in mean Horowitz index values between the groups

time	non-POC group	POC group	t test		
			difference	95% CI	*p*-value
0	292.83	346.19	53.36	(5.91, 100.82)	0.028
24	350	395.61	45.61	(8.29, 82.93)	0.017
48	326.72	385.26	58.54	(16.06, 101.01)	0.008
72	308.03	402.87	94.84	(60.18, 129.51)	< 0.001

*Abbreviations*: *POC* point of care, *CI* confidence interval

### Norepinephrine dosage and albumin serum levels evaluation

The maximum single dose of norepinephrine (µg/kg/min) administered to every patient in both groups during the first 24 h was recorded. In the non-POC group and POC group, the maximum doses of norepinephrine were 0.379 and 0.193, respectively (*p* < 0.001, difference between the means: 0.186, 95% CI: 0.105–0.267). Serum albumin levels (g/l) in both groups were measured before and after LuTx. There was no significant difference in preoperative mean serum albumin levels between POC group and non-POC group (44.43 vs 44.19; *p* = 0.84, difference between means: 0.24, 95% CI: (-2.11)-2.58). The mean serum albumin levels after LuTx surgery were significantly higher in the POC group than in the non-POC group (41.55 vs 29.37), with *p* < 0.001, difference between means 12.18 and 95% CI: 9.81–14.55.

### Postoperative mechanical ventilation duration and length of ICU stay and 1-year survival

**Fig. 5:**
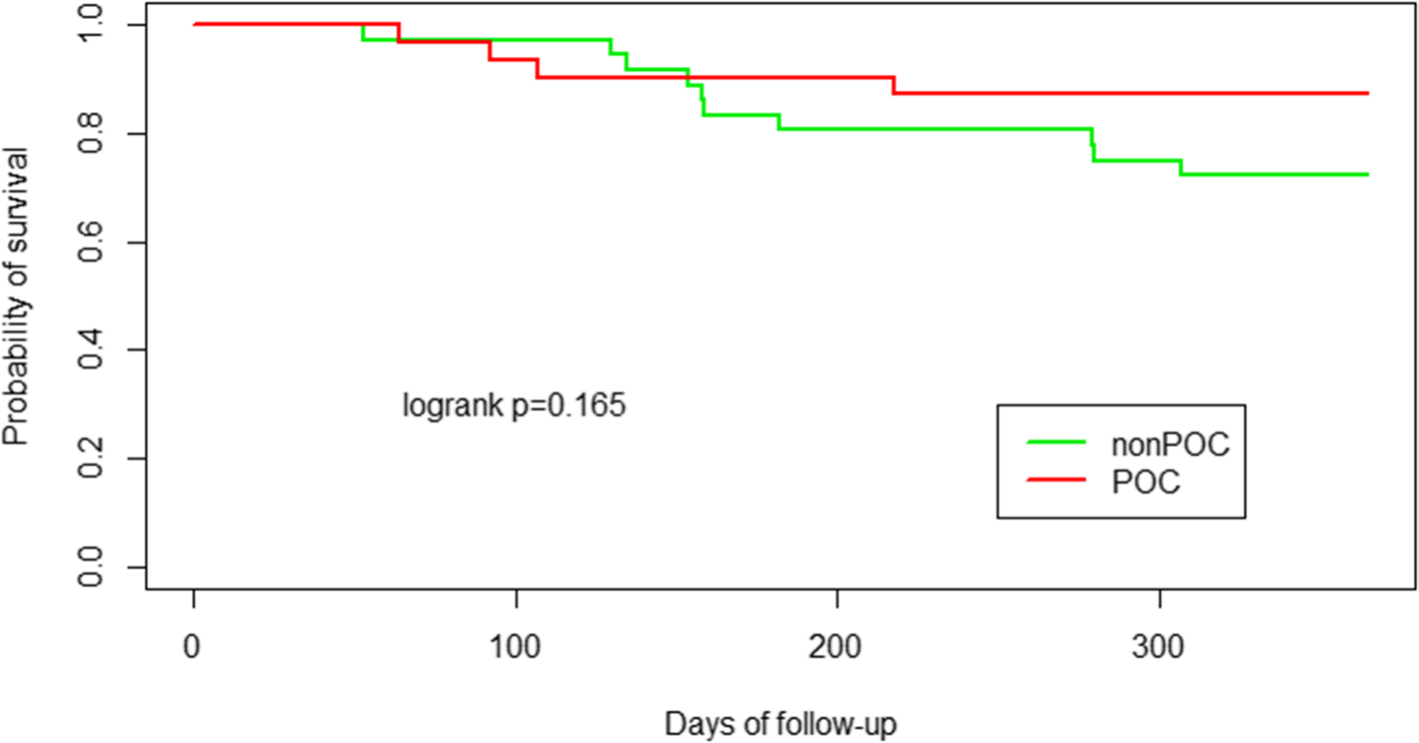
Kaplan Meier survival curve for patients in non-POC group (green line) vs POC group (red line). Abbreviations: POC: point of care

Duration of mechanical ventilation and length of ICU stay after LuTx surgery were decreased in POC group. However, this difference did not cross the boundary for statistical significance as shown in Table 5. During the 1-year follow-up study period after LuTx, more patients died in the non-POC group than in the POC group, although the difference in 1-year survival was not statistically significant (10 patients in non-POC group vs. 4 patients in POC group; *p* = 0.17). In both groups, 30-day mortality was 0%. In POC group, 90-day mortality was 3.2% (*n* = 1, patient with cystic fibrosis that died at day 64 because of fulminant Burkholderia cenocepacea infection). In non-POC group 90-day mortality was 2,8% (*n* = 1, patient with pulmonary fibrosis died at day 53 because of bronchopneumonia due to Pseudomonas aeruginosa). After 90-postoperative day, other causes of death during first year were infection (8 patients) and cardio-renal failure (1 patient) in non-POC group and infection (1 patient), pancreatic cancer (1 patient) and brain stroke (1 patient) in POC group. A Kaplan–Meier 1-year survival curve is shown in Fig. 5.

**Table 5:** Duration of mechanical ventilation and length of intensive care unit stay after LuTx surgery in non-POC vs POC group

	non-POC group			POC group			Wilcoxon test
	mean	median	IQR	mean	median	IQR	*p*-value
MV (hours)	147.8	35.5	50.5	90.3	25	36	0.17
ICU stay (days)	13	6	3.3	9.5	5	4	0.27

*Abbreviations*: *ICU* intensive care unit, *IQR* interquartile range, *LuTx* lung transplantation, *MV* mechanical ventilation, *POC* point of care

## Discussion

PGD negatively contributes to increased short-term and long-term morbidity and mortality after LuTx [8, 9]. While the exact pathogenesis is not completely understood, multiple risk factors are associated with the development of PGD including donor-specific and recipient-specific variables [23]. Additionally, postoperative risk or complicating factors such as hypotension, fluid overload, vascular anastomotic complications, inadequate mechanical ventilation, and pneumonia have been reported to contribute to PGD [6]. Finally, intraoperative anesthetic management has been reported to have a potentially significant influence on the development of PGD [10].

Ischemia–reperfusion injury after lung allograft implantation has been shown to lead to PGD development [24]. Interestingly, the pulmonary endothelial glycocalyx is particularly prone to ischemia–reperfusion injury and shedding of the glycocalyx has been linked to respiratory failure and the development of ARDS in mice [25]. The control of this reperfusion has been theorized as a method of attenuating the development of PGD in lung transplantation, and the utilization of VA ECMO for intraoperative support has been described as a method to accomplish this control. Hoetzenecker et al. demonstrated that intraoperative VA ECMO support provides optimal reperfusion conditions that translate into superior graft function [22]. Although routine use of intraoperative ECMO is generally advocated, there is still a non-negligible risk of undesirable bleeding associated with this method. Thomas et al. noted that achieving the optimal anticoagulation balance to prevent bleeding and thrombosis in ECMO patients is extremely complex, and experts in hemostasis should be a part of an institutional ECMO team and continuously available for immediate management [26].

Transfusion of a large amount of blood products, especially FFP, to manage intraoperative blood loss during LuTx is an independent risk factor for PGD through transfusion-related acute lung injury (TRALI) [27–29]. Diamond et al. reported that the prevalence of greater than 1 L RBC intraoperative transfusion was 34%, and in the adjusted analysis, this was associated with a nearly twofold increased risk for the development of PGD grade 3 [30]. In addition, apart from the abovementioned TRALI, blood product transfusion alone is associated with transfusion-associated circulatory overload (TACO), pulmonary infections and prolonged ICU stays [31]. The incidence of TACO is reported to be highest after the transfusion of FFP, followed by RBCs and platelets [32]. Perioperative POC-targeted coagulopathy management reduces the amount of blood transfusion products needed [33]. We have previously demonstrated that this perioperative approach practically eliminated the need for FFP transfusion in the POC study group during LuTx surgery [18]. This is of particular interest regarding avoidance of the FFP-associated volume expansion effect, which may negatively contribute to PGD development. Despite the abovementioned reduction in blood product transfusion, a certain amount of fluid is necessary to maintain normovolemia during surgery. However, excessive perioperative crystalloid and colloid administration might be associated with fluid overload and therefore increase the risk of PGD development.

In our study, 5% albumin solution was used solely as volume replacement therapy in the POC group. Albumin is a medium-sized molecule with a molecular weight of 66–69 kDa and is the most abundant protein in human plasma (40 g/l out of a total of 70 g/l). Albumin is synthesized exclusively in the liver and plays an important role in numerous processes. For example, it serves as a major extracellular antioxidant and a major transporter in plasma, responsible for 75% of oncotic plasma pressure. Therefore, albumin solution is considered to be the standard colloidal resuscitation fluid [34]. Another crucial role of albumin appears to be its positive effect on the physiological part of the endothelial glycocalyx where it maintains a functioning vascular barrier, especially in patients where increased capillary leakage is present [35, 36]. This typically occurs during LuTx as a part of ischemia–reperfusion-induced lung graft injury or as a part of systemic inflammatory response syndrome (SIRS) aggravated by the ECMO circuit.

Fluid management during all types of surgical procedures affects postoperative outcomes [37]. Inadequate fluid management may be associated with mitochondrial dysfunction and the promotion of inflammation, which can lead to decreased lung allograft function [12, 25]. However, the use of colloids in volume replacement therapy remains a subject of debate. Uhlig et al. reviewed and performed a meta-analysis of 3 randomized controlled trials comparing albumin versus crystalloid solutions for intravascular volume expansion in critically ill patients with ARDS and based on the findings of their review, colloid therapy with albumin improved oxygenation but did not affect mortality [19]. Torres et al. studied the effect of different kinds of fluid administration on the vascular endothelium and microcirculation and found that the administration of protein-rich solutions such as albumin helped to rebuild the endothelial glycocalyx [38]. Mendes et al. conducted similar investigations in a rat model of acute lung injury (ALI), and their results revealed that both iso-oncotic and hyper-oncotic albumin solutions were associated with decreased lung injury as compared to Ringer’s lactate [39]. Moreover, Moreno Garijo et al. described the importance of albumin as a primary fluid at Toronto Lung Transplant Program [40]. However, the data supporting the intraoperative albumin utilization in their review were lacking.

In our study, targeted coagulopathy management and 5% albumin solution administered exclusively as volume replacement therapy during LuTx surgery resulted in significant improvement in lung allograft function in the first postoperative 72 h in the POC study group compared to the non-POC study group measured by Horowitz index. This intervention also resulted in significant decrease of PGD grade 2–3 at time point 72 in POC group. This is of particular interest, as most studies examine the incidence of PGD grade 3 at 72 h. However, in our study there was no statistically significant difference in occurrence of PGD grade 3 between the non-POC and POC group at all tracked time points. Additionally, the mean value of the maximum norepinephrine level during the first 24 h after LuTx was found to be significantly decreased in the POC group. This finding supports the theory that albumin as volume replacement therapy during LuTx surgery may provide greater hemodynamic circulatory stability in the POC group during the first 24 h after surgery through both volume replacement and its hypothesized anti-inflammatory effect on the reduction in SIRS [34]. Our data from secondary analysis suggest that administration of 5% albumin during LuTx surgery may have a more protective effect on shedding of the glycocalyx and therefore reduce vasoplegia and SIRS. Moreover, significantly higher postoperative levels of serum albumin in the POC group may further contribute to postoperative better graft function and circulatory stability through the abovementioned mechanisms.

Our study has several limitations that require rigorous and transparent discussion. First, a major limitation is that our study design contained two interventions in one study protocol (targeted coagulopathy management and 5% albumin in POC study group). As targeted coagulopathy management led to decrease of blood loss and blood products transfusion, the study was preliminarily terminated by the institutional review board due to positive results in favor of the POC approach. This preliminary termination resulted in a relatively small cohort size in each group, precluding further evaluation of effect of the second intervention in the study (5% albumin administration). Moreover, two interventions in one study protocol limits our ability to identify the precise extent of how either the first or second intervention contributed to the study results.

Heterogeneity in the patient age distribution between the non-POC group, where patients were older, versus the POC group that contained a greater proportion of younger patients with cystic fibrosis was another limitation. This population difference is important to highlight, as a variety of etiology-based comorbidities can impact intraoperative management and outcomes [41]. In particular, it is generally accepted that LuTx outcomes are better in younger patients with cystic fibrosis. However, a recently reported study by Fessler et al. demonstrated a higher perioperative utilization of RBCs and FFP in patients with cystic fibrosis compared to those with chronic obstructive pulmonary disease or pulmonary fibrosis [42]. Therefore, despite an imbalance between population age and etiology of end-stage lung disease, intraoperative targeted coagulopathy management together with 5% albumin administration significantly reduced blood loss and blood product transfusion in the POC group [18]. A final limitation is focused on the completeness of our preoperative laboratory evaluation. Preoperative serum albumin levels in 15 patients (9 patients in the non-POC group and 6 patients in the POC group) were not measured for technical reasons; however, the postoperative serum albumin levels records were complete for all patients in the study cohort.

To the best of our knowledge, despite abovementioned limitations, the research presented herein represents the first clinical trial attempting to investigate the effect of the perioperative use of targeted bleeding and coagulopathy management combined with 5% albumin administration on lung allograft function after LuTx. Furthermore, our data provide a level of evidence suggesting albumin as an optimal choice for intraoperative resuscitation in lung transplantation that to date has been based on expert opinion in the literature. However, further investigation in this area is highly needed to provide deeper insight into potential beneficial effect of perioperative use of 5% albumin solely as volume replacement therapy during LuTx on PGD incidence. The authors suggest design future trial with 5% albumin solution administrated intraoperatively as the only intervention in study group.

## Conclusions

The results of this study indicate that targeted coagulopathy management and 5% albumin solution solely used as volume replacement therapy during LuTx surgery may improve early lung allograft function, provide better circulatory stability during the early post-operative period, and have potential to decrease the incidence of PGD without negative effect on 1-year survival. However, further investigation is highly needed to provide deeper insight into mechanisms of potential beneficial effect of perioperative use of 5% albumin solely as volume replacement therapy during LuTx on PGD incidence, CLAD development, and long-term outcomes.

## Data Availability

All data generated or analyzed in the current article are available from the corresponding author on reasonable request.

## Abbreviations

ACR: Acute cellular rejection
ALI: Acute lung injury
AMR: Antibody mediated rejection
ARDS: Acute respiratory distress syndrome
CI: Confidence interval
CLAD: Chronic lung allograft dysfunction
DCD: Donation after circulatory death
ECMO: Extracorporeal membrane oxygenation
FFP: Fresh frozen plasma
GERD: Gastroesophageal reflux disease
ICU: Intensive care unit
ISHLT: International Society for Heart and Lung Transplantation
LuTx: Lung transplantation
PFA: Platelet function analyzer
PGD: Primary graft dysfunction
POC: Point of care
RBCs: Red blood cells
ROTEM: Rotational thromboelastometry
SIRS: Systemic inflammatory response syndrome
TACO: Transfusion-associated circulatory overload
TEE: Transesophageal echocardiography
TRALI: Transfusion-related acute lung injury
VA ECMO: Veno-arterial extracorporeal membrane oxygenation
